# Iron oxide@chlorophyll clustered nanoparticles eliminate bladder cancer by photodynamic immunotherapy-initiated ferroptosis and immunostimulation

**DOI:** 10.1186/s12951-022-01575-7

**Published:** 2022-08-11

**Authors:** Yu-Cheng Chin, Li-Xing Yang, Fei-Ting Hsu, Che-Wei Hsu, Te-Wei Chang, Hsi-Ying Chen, Linda Yen-Chien Chen, Zi Chun Chia, Chun-Hua Hung, Wu-Chou Su, Yi-Chun Chiu, Chih-Chia Huang, Mei-Yi Liao

**Affiliations:** 1 Department of Photonics, National Cheng Kung University, Tainan, 70101 Taiwan; 2 Department of Biological Science and Technology, China Medical University, Taichung, 406 Taiwan; 3 Division of Urology, Department of Surgery, Taipei City Hospital Zhongxing Branch, Taipei, 103 Taiwan; 4 Division of Urology, Department of Surgery, Taipei City Hospital Heping Fuyou Branch, Taipei, 100 Taiwan; 5 Department of Applied Chemistry, National Pingtung University, Pingtung, 900 Taiwan; 6 Nanofabrication Laboratory, Department of Electrical and Computer Engineering, University of Canterbury, Christchurch, New Zealand; 7 Center of Applied Nanomedicine, National Cheng Kung University, Tainan, 70101 Taiwan; 8 Department of Urology, College of Medicine and Shu-Tien Urological Research Center, National Yang Ming Chiao Tung University, Taipei, 112 Taiwan; 9 Department of Exercise and Health Sciences, University of Taipei, Taipei, 100 Taiwan; 10 Core Facility Center, National Cheng Kung University, 70101 Tainan, Taiwan

## Abstract

The escape of bladder cancer from immunosurveillance causes monotherapy to exhibit poor efficacy; therefore, designing a multifunctional nanoparticle that boosts programmed cell death and immunoactivation has potential as a treatment strategy. Herein, we developed a facile one-pot coprecipitation reaction to fabricate cluster-structured nanoparticles (CNPs) assembled from Fe_3_O_4_ and iron chlorophyll (Chl/Fe) photosensitizers. This nanoassembled CNP, as a multifunctional theranostic agent, could perform red-NIR fluorescence and change the redox balance by the photoinduction of reactive oxygen species (ROS) and attenuate iron-mediated lipid peroxidation by the induction of a Fenton-like reaction. The intravesical instillation of Fe_3_O_4_@Chl/Fe CNPs modified with 4-carboxyphenylboronic acid (CPBA) may target the BC wall through glycoproteins in the BC cavity, allowing local killing of cancer cells by photodynamic therapy (PDT)-induced singlet oxygen and causing chemodynamic therapy (CDT)-mediated ferroptosis. An interesting possibility is reprogramming of the tumor microenvironment from immunosuppressive to immunostimulatory after PDT-CDT treatment, which was demonstrated by the reduction of PD-L1 (lower “off” signal to the effector immune cells), IDO-1, TGF-β, and M2-like macrophages and the induction of CD8^+^ T cells on BC sections. Moreover, the intravesical instillation of Fe_3_O_4_@Chl/Fe CNPs may enhance the large-area distribution on the BC wall, improving antitumor efficacy and increasing survival rates from 0 to 91.7%. Our theranostic CNPs not only demonstrated combined PDT-CDT-induced cytotoxicity, ROS production, and ferroptosis to facilitate treatment efficacy but also opened up new horizons for eliminating the immunosuppressive effect by simultaneous PDT-CDT.

**Supplementary Information:**

The online version contains supplementary material available at 10.1186/s12951-022-01575-7.

## Introduction

It has been reported that bladder cancer (BC), one of the most common cancers worldwide in humans, is increasing yearly, with an estimate of over 550,000 new cases each year [1]. The evasion of immune surveillance and programmed cell death are two important factors that promote BC progression. Despite the utility of active intervention by transurethral resection and intravesical bacillus Calmette-Guérin (BCG) [2, 3], which could partially suppress BC progression, 20% of patients still developed muscle-invasive BC and were insensitive to monotherapy [4]. Unfortunately, programmed death-ligand 1, a coinhibitory factor of the immune response, is highly expressed in BC, resulting in an immunosuppressive “cold tumor” environment that diminishes immunotherapy efficacy [5]. In summary, the limitation of treatment options and elevation of recurrence potential are due to restricted immune cell infiltration, poor response to chemotherapy, and the adverse effects of long-term treatment [3, 6]. Therefore, designing a multitarget treatment strategy could provide new insights into eradicating BC progression. Recently, there has been widespread interest in the development of new theranostic nanoagents for exploring a noninvasive treatment modality for BC, which could enhance detection and therapeutic efficiency because the unique crosstalk of the cellular response to active uptake, penetration, and circulation relies on the size and shape effects of small particles [4, 7–13]. The extensive surface chemistry of nanoparticles as a result of surface modification, in contrast to the complicated synthesis of organic derivatives, is beneficial for the targeted delivery of drugs to achieve high accumulation in a lesion and prevent clearing by the reticuloendothelial system [8, 9, 12]. This study proposes new multifunctional nanoparticle (NP) agent that (1) enhances delivery efficacy to achieve large-area distribution by minimally invasive therapeutic techniques, (2) embeds lipid peroxidation activator adjuvants to interrupt redox balance by reactive oxygen species (ROS) induction, and (3) loads nano-photosensitizers for combined photodynamic therapy (PDT) and chemodynamic therapy (CDT) to reprogram the tumor microenvironment (TME) by reducing the immune escape effect.

PDT combined with CDT has recently become important in strategies to kill cancer cells with minimal side effects by inducing the generation of ROS through modulating the cellular environment [13–21]. On the other hand, PDT may not only ablate tumor cells by heat but also show potential to initiate an antitumor immune response by triggering immunogenic cell death (ICD) [22, 23]. Thermal ablation through PTT is an efficient therapeutic route to remove cancer lesions [10, 24], but the poor thermal control and high laser power-induced heat injury to normal tissues are significant concerns in clinical use [25]. PDT utilizes lower laser power to induce localized ROS generation and prevent heat damage to surrounding normal tissues. However, most of the photosensitizers used in PDT have potential toxicity [26, 27] and poor water solubility, which greatly affects their therapeutic efficiency [28]. To achieve superior tumor control, whether PDT can be combined with immune manipulation to reverse the tumor immunosuppressive microenvironment (TIME) is another important issue.

Indeed, CDT includes a selective tumor-specific reaction that can be applied with PDT to efficiently enhance therapeutic efficacy in several neoplasms [20, 21]. The integration of peroxidase mimics with transition metal ions can generate highly reactive hydroxyl radicals and trigger a decrease in intracellular GSH levels in other malignancies by Fenton-like reactions [17–19]. However, the use of ferroptosis-related CDT relying on the metal-dependent reaction [14–16] has commonly shown limited efficiency due to the consumption of H_2_O_2_ in the tumor site, with a high [oxidized metal ion]/[reduced metal ion] ratio [29, 30] that decreases the catalytic rate and results in unsatisfactory efficacy in eradicating the primary tumor. In addition, the highly efficient delivery and accumulation of metal-based species in malignant lesions through blood vessels was the first goal of specific CDT induction [31]. Nevertheless, systemic administration usually leads to unfavorable organ accumulation that may cause underlying chronic metal poisoning or tissue damage [32].

**Scheme 1:**
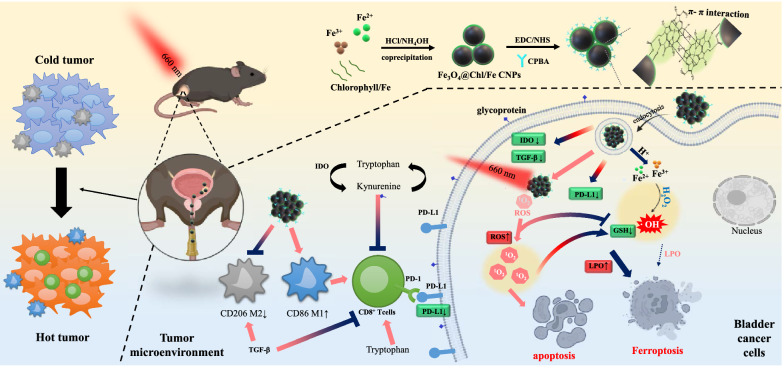
Schematic illustration of the synthesis of Fe_3_O_4_@Chl/Fe-CPBA CNPs for targeted delivery into BC cells and demonstration of their antitumor effect and immunoregulatory effect within BC cells after the combination of chemodynamic and photodynamic therapy

PDT was combined with other endogenous chemical therapeutic strategies to improve treatment efficiency in malignant BC [6, 14–16, 33–35]. For example, O_2_-generating nanoparticles (iron oxide@organoselenium [33] and HSA-MnO_2_-Ce6 [34]) and chemotherapeutic Pt released from iron oxide/Pt hybrid NPs [35] can exert some effect on bladder tumors when the therapeutic outcome is not satisfactory and can even promote PDT-induced cytotoxicity and antitumor immunity. These designed nanoagents are relatively complex building blocks, and there is still room for improvement. An effective strategy of combining PDT-CDT nanoagents with a simple one-pot synthesis design to eradicate BC remains a challenge and has not been reported until now. Herein, we demonstrated a green and one-pot synthesis process by the nucleation and growth of Fe_3_O_4_ nanocrystals along with a chlorophyll-Fe(II) (Chl/Fe) matrix to form cluster-structured nanoparticles (CNPs), denoted Fe_3_O_4_@Chl/Fe CNPs. The Chl/Fe molecule is a reduced-state Fe^2+^ and acts as a PDT/CDT adjuvant. They are clustered by π–π interactions between the Chl molecules stacked on the Fe_3_O_4_ CNP surface (Scheme 1). The iron species from the Fe_3_O_4_@Chl/Fe CNPs could provoke peroxidase-like activity to generate hydroxyl radicals by decomposing H_2_O_2_ prior to PDT. They could thus evoke the lipid peroxidation process of the CDT reaction after the consumption of the antioxidant GSH in cancer cells (Scheme 1). Conjugation with the specific targeting molecule carboxyphenylboronic acid (CPBA) can increase the delivery efficacy of Fe_3_O_4_@Chl/Fe catalysts to enhance the rate of CDT generation by the Fenton reaction and to reduce the antioxidant capacity of cancer cells, sensitizing these cells to subsequent PDT treatment and increasing immune-mediated elimination by downregulating PD-L1 expression and inhibiting immunosuppressive cell accumulation after CDT–PDT treatment. Inspired by the transurethral BCG treatment process, in which BCG is instilled into the bladder through a catheter, rather than systemic circulation by intravenous injection, we used intravesical instillation of nano-photosensitizers that can interact directly with tumors in the bladder (Scheme 1). Our CDT–PDT treatment in BC revealed the first immunostimulatory feature in the irradiated area of light [6, 8, 36, 37], which reprograms the tumor microenvironment from an immunosuppressive “cold” to an immunogenic “hot” tumor. CDT–PDT treatment may not only suppress the production of immunosuppressive factors and cells but also boost the accumulation of immunostimulatory cells. Thus, the intracellular and local amplified oxidative stress and additive anti-immunity effect improved the survival rate from 0 to 91.7% and minimized the adverse impact of systemic management.

## Materials and methods

Iron(II) chloride tetrahydrate (FeCl_2_·4H_2_O, 99–102%; J. T. Baker), Iron(III) chloride hexahydrate (FeCl_3_·6H_2_O; Merck), Hydrochloric acid (HCl, 37%; Fluka), Ammonia solution (25%,Merck), sodium iron chlorophyllin salt (Chl/Fe), H_2_N-PEG-NH_2_ (MW = 3500; JenKem Technology), 1-ethyl-3-(-3-dimethylaminopropyl)carbodiimide hydrochloride (EDC, 99%; Matrix scientific), *N*-hydroxysuccinimide (NHS, 98%+; Alfa Aesar), 4-carboxyphenylboronic acid (HO_2_CC_6_H_4_B(OH)_2_; Aldrich), hydrogen peroxide (H_2_O_2_, 34.5–36.5%, Sigma-Aldrich), 3,3′,5,5′-Tetramethylbenzidine (TMB, 99%, Sigma-Aldrich) 3-(4,5-dimethylthiazol-2-yl)-2,5-diphenyltetrazolium bromide (MTT assay reagent, GoldBio), 2,7-dichlorofluorescin diacetate (DCFH-DA, ≥ 97% Sigma-Aldrich), transferrin (≥ 98%, Sigma-Aldrich), Arginylglycylaspartic acid (RGD, ≥ 97% Sigma-Aldrich), *N*,*N*-dimethyl-4-nitrosoaniline (RNO, 98%, Alfa Aesar), Imidazole (99%, Acros organics), dimethyl sulfoxide (DMSO, ≥ 99.7%, Fisher scientific), Cytotoxicity LDH Assay Kit purchased from dojindo, Glutathione Fluorescent Detection Kit purchased from ThermoFisher, Cell medium (DMEM, RPMI-1640, McCoy’s 5A, F12K) purchased from Corning.

### Fe_3_O_4_@Chl/Fe synthesis (coprecipitation method)

FeCl_3_·6H_2_O (0.540 g) and 200 mg of Chl/Fe in 2 mL of HCl were gently stirred for 1 h at room temperature. Afterward, 0.5 mL of a solution containing 0.1988 g of FeCl_2_·4H_2_O in concentrated HCl was added to the solution, followed by dropwise addition of 20 mL of NH_4_OH (2 M). The solution color gradually changed from dark orange to black, indicating the formation of Fe_3_O_4_@Chl/Fe nanoclusters. After completion of the reaction, Fe_3_O_4_@Chl/Fe was centrifuged at 9500 rpm for 10 min and redispersed with D.I. water more than three times. The highly dispersed product was collected from the supernatant after centrifugation for another 10 min at 3000 rpm.

### Fe_3_O_4_@Chl/Fe-PEG-modified molecules synthesis

4 mg of NH_2_-PEG-NH_2_ was mixed with 200 µL of synthesized Fe_3_O_4_@Chl/Fe with desired concentration. The mixture was ultrasonicated in the dark for 5 min to form Fe_3_O_4_@Chl/Fe-PEG. 1.5 mg of NHS and 1.5 mg of EDC were mixed into 0.1 mL of deionized water. The two mixtures were then mixed with an addition of 0.6 mL deionized water. The sample was then added with different molecules, such as CPBA (0.05 mM, 50 µL), arginylglycylaspartic acid (RGD peptide, 60 µg/mL, 50 µL), Transferrin (60 µg/mL, 50 µL), for molecule modification, followed by 5 min of ultrasonication. 200 µL of synthesized Fe_3_O_4_@Chl/Fe-PEG was mixed with the prepared mixture and ultrasonicated in the dark at 4 °C for 1 h. The sample was then centrifuged at 9500 rpm for 10 min before re-dissolving the centrifuged material into 200 µL DI water to obtain Fe_3_O_4_@Chl/Fe-PEG-modified molecules.

### Singlet oxygen detection

Samples with different concentrations were placed under the LED board and exposed at the 75 mW/cm^2^ power rating. RNO (1 mM/10 µL) and imidazole (1 mM/10 µL) were used as singlet oxygen detection indicators. The absorption peak of RNO at 440 nm was measured at 0 and 30 min of exposure time.

### In vitro characterization of chromogenic performance

TMB was used as an indicator for kinetic analysis via colorimetry to investigate the catalysis of the Fenton reaction. The examination of peroxidase-like activity was carried out in a 4 mL quartz cell with 2.28 mL of an aqueous solution containing 0.5 ppm_[Fe]_ Fe-based samples and TMB (0.8 mM). Then, 120 µL of 20 mM and 2 M hydrogen peroxide solutions were added to the above-mixed solution, resulting in final concentrations of 1 and 100 mM, respectively. The absorption peak of TMB at 652 nm was measured every 10 s and recorded over time.

### Temperature examination under 650 and 808 nm laser irradiation

Fe_3_O_4_@Chl/Fe solution (100 ppm_[Fe]_) placed in 96 well plates was irradiated using 650 and 808 nm continuous wave (CW) lasers, respectively. A thermocouple was immersed in the material solutions to determine the temperature elevation. The power density of 650 and 808 nm lasers was 210 and 195 mW/cm^2^, respectively. The thermal curves of these solutions were monitored from 0 to 10 min every 10 s using a thermometer.

### In vitro cell viability measurement

Hela, T24, MB49, 3T3, VERO, and SV-HUC1 cells were seeded in the 96-well cell culture plate (8000 cells/well) overnight, treated with 100 µL of DMEM, McCoy’s 5A, RPMI, and F-12K media containing different concentrations of Fe_3_O_4_@Chl/Fe and Fe_3_O_4_@Chl/Fe-ligand (0.2, 1, 5, 10, 50, and 100 ppm) for 1 h and maintained in 37 °C incubator with 5% CO_2_. After removing the media containing Fe_3_O_4_@Chl/Fe and Fe_3_O_4_@Chl/Fe-ligand, new media was added. The 96-well cell culturing plate was then exposed under a 660 nm LED light with a 75 mW/cm^2^ power rating for 10 min, while the controlled group was maintained in the dark. After another 24 h, 100 µL of MTT reagent (1.2 mM) was added to both control and test groups. The samples were then placed in an incubator for another 1 h to obtain purple crystals formed by the reaction between the assay and the mitochondrion of live cells. The purple crystals were dissolved with dimethyl sulfoxide (DMSO), measured with UV–Vis spectrometry under 565 nm absorbance, and detected the viability of cells.

### Cellular uptake measurement

Human bladder carcinoma cells (T24) and mouse bladder carcinoma cells (MB49) were cultured in McCoy’s 5A and RPMI media containing 10% of fetal bovine serum (FBS) and 1% of penicillin (P/S) in a 5% CO_2_-filled incubator at 37 °C. T24 and MB49 cells (1 × 10^4^ cells), Fe_3_O_4_@Chl/Fe (20 ppm), and Fe_3_O_4_@Chl/Fe-CPBA (20 ppm) were cultured together in a 3.5 cm culture dish for 24 h in the dark. The chamber slide was rinsed with PBS 3 times after incubation. The cells were harvested with 0.25% trypsin and were centrifuged at 1000 rpm for 10 min; then, the supernatant was removed. The precipitated cells were dissolved in 100 µL DI water and sonicated for 15 min. Finally, the suspensions were centrifuged at 14,000 rpm for 10 min, the residue was extracted, and the supernatant was dissolved in 4.5 M HCl. The concentration of iron was determined by atomic absorption spectroscopy.

### In vitro ROS assessment

The in vitro ROS generation was determined by the fluorescence change resulting from the oxidation of DCFH-DA. The T24, SV-HUC1, and MB49 cells were treated with Fe_3_O_4_@Chl/Fe and Fe_3_O_4_@Chl/Fe-CPBA for 1 h; new media was added after removing the media containing nanoparticles. The culture dish was then exposed to a 660 nm LED light with a 75 mW/cm^2^ power rating for 1 min while the controlled group was maintained in the dark, then DCFH-DA (10 µM) in the serum-free medium was added. After 30 min co-incubation, the cells were washed with PBS before fluorescence microscope observation.

### In vitro lipid peroxidation analysis

BODIPY® 581/591 C11 (4,4-difluoro-5-(4-phenyl-1,3-butadienyl)-4-bora-3a,4a-diaza-s-indacene-3-undecanoic acid) was used to detect ROS in cells and membranes. The T24, SV-HUC1, and MB49 cells were treated with Fe_3_O_4_@Chl/Fe and Fe_3_O_4_@Chl/Fe-CPBA for 1 h; new media was added after removing the media containing nanoparticles. The culture dish was then exposed to a 660 nm LED light with a 75 mW/cm^2^ power rating for 1 min, while the controlled group was maintained in the dark, then BODIPY® 581/591 C11 (10 µM) in the serum-free medium was added for 40 min of staining time. After co-incubation for 20 min, the cells were washed with PBS before fluorescence microscope observation.

### Western blotting assay

T24 and MB49 cells (10^6^ per dish) subjected to the indicated treatments were harvested and lysed in lysis buffer (M-PERTM mammalian protein extraction reagent, Thermo Fisher Scientific Inc.) for 30 min on ice and then centrifuged at 14,000 rpm for 20 min at 4 ℃ to remove precipitates. The obtained proteins were adjusted to equal loads (20 µg per well). They were separated through electrophoresis on a 12% sodium dodecyl sulfate-polyacrylamide gel and subsequently transferred to a 0.45 μm polyvinylidene difluoride (PVDF) membrane, blocked using 5% skim milk, and immunoblotted using anti-GPX4 (cat no. #ab125066, 1:3000 dilutions, Abcam plc.) and GAPDH (cat no. #2118, 1:5000 dilutions, Cell Signaling Technology Inc.) monoclonal antibodies. The membranes were then washed using Tris-buffered saline supplemented with 0.1% Tween-20 and incubated again with horseradish peroxidase (HRP)-conjugated secondary antibody (cat no. #7074, 1:5000 dilutions, Cell Signaling Technology Inc.). The corresponding bands were detected using HRP substrate (Merck Millipore) and captured using an imaging system (UVP Bio-Spectrum; Analytik Jena US LLC, Upland, CA). The images were analyzed by ImageJ software (NIH, Bethesda, Maryland) for protein expression normalization and quantification.

### PD-L1 and IDO-1 detection assay

T24 cells (2 × 10^5^ per well) subjected to the indicated treatments were harvested, fixed with 4% paraformaldehyde for 30 min and then washed by centrifugation with PBS. The cells were permeabilized by adding ice-cold 100% methanol slowly to prechilled cells to a final concentration of 90% methanol under gentle vortexing. The cells were then hybridized with anti-PD-L1 (cat no. #13684, 1:400 dilutions, Cell Signaling Technology Inc.) or permeabilized before staining with anti-IDO-1 (cat no. #11650-T58, 1:400 dilutions, Sino Biological Inc.) primary antibody for 1 h followed by FITC-conjugated secondary antibody for another 1 h after washing with PBS 3 times. The prepared cells were measured by flow cytometry (FACSCanto II; BD Biosciences) (excitation wavelength: 488 nm; emission wavelength: 515–545 nm), and the results were analyzed using FlowJo software (BD Biosciences).

### In vivo animal experiments

Female C57BL/6 mice (12 weeks old) were purchased from the Laboratory Animal Center, Medical College, National Cheng Kung University, Tainan, Taiwan. All research protocols were approved by the Institutional Animal Care and Use Committee (IACUC) of the Animal Experiment Center of Cheng Kung University. An orthotopic bladder cancer-bearing mice model was established by in situ injection with MB49 cancer cells (1 × 10^7^ cells suspended in 100 µL of PBS) into the bladder wall.

### In vivo ferroptosis and photodynamic therapy

The C57BL/6 tumor-bearing mice were randomly divided into 5 groups and treated with (1) in situ injections of PBS on Days 0 and 7, (2) in situ injections of Fe_3_O_4_@Chl/Fe (100 ppm_[Fe]_) for 1 h on Days 0 and 7, (3) 660 nm laser irradiation 1 h after in situ injections of Fe_3_O_4_@Chl/Fe (100 ppm_[Fe]_) on Days 0 and 7, (4) in situ injections of Fe_3_O_4_@Chl/Fe-CPBA (100 ppm_[Fe]_) for 1 h on Days 0 and 7 or (5) 660 nm laser irradiation 1 h after in situ injections of Fe_3_O_4_@Chl/Fe-CPBA (100 ppm_[Fe]_) on Days 0 and 7. The laser treatment groups were irradiated by the laser at 75 mW/cm^2^ for 10 min. The tumor growth and body weight of each mouse were recorded every 7 days and 2 days, respectively. The tumor area of each mouse was measured by ultrasound (US) and analyzed by ultrasonic software (VisualSonics). On the 14th day post-injection, the mice were sacrificed, and the major organs (heart, liver, spleen, lungs, and kidneys) and tumors were isolated and preserved in a 4% paraformaldehyde solution for histological analysis.

### Histological examination

Histological analysis of mice tissues was performed after 14 days of treatment. Major organs (heart, liver, spleen, lungs, and kidneys) and tumors were performed with hematoxylin and eosin stain. The H&E stained sections were then visualized by microscope (at 20× objective).

### Immunohistochemistry (IHC)

Tumors extracted from each group were fixed, embedded, sliced, and stained with IHC reagent (DAB500 Merck Millipore). The IHC staining process was performed according to the instructions in the Millipore datasheet. IHC staining-related primary antibodies included CD8 (cat no. #10980-T48, 1:200 dilution, Sino Biological), CD86 (cat no. #19589, 1:200 dilution, Cell Signaling Technology), CD206 (cat no. #ab64693, 1:200 dilution, Abcam), IDO-1 (cat no. #11650-T58, 1:200 dilution, Sigma-Aldrich), and TGF-β (cat no. # SAB5300197, 1:200 dilution, Sigma-Aldrich). Images were captured by a Nikon ECLIPSE Ti-U microscope at 200× magnification and quantified by ImageJ software.

### Instruments

Transmission electron microscopy (Hitachi 7500 and JEM-2100F) was used to determine the structures of the nanomaterials. The Fe_3_O_4_@Chl/Fe CNPs solution on a grid coated with a hole-containing carbon support film was blotted dry with filter paper to leave a thin film of particle suspension in the wells. Immerse the grid in liquid ethane cooled by liquid N_2_. Fluorescence spectrum (Biotek Synergy H1) and UV–Visible Spectrophotometer (JASCO V-730, Japan) were used to measure the fluorescence and absorption of the Fe_3_O_4_@Chl/Fe-related samples. The particle sizes and zeta potentials (Horiba SZ-100, Japan) measured the samples dispersed in an aqueous solution. Thermogravimetric analysis (TGA, TA-Q50, USA) and AAS (SensAA GBC, Australia) were utilized for measuring the organic and metal composites of Fe_3_O_4_@Chl/Fe nanoparticles. FT-IR spectrometer (JASCO FT/IR-4700), X-ray diffractometer (XRD, Bruker D8 Discover, Karlsruhe, Germany), and Raman spectra were performed to analyze the surface and crystal structures of samples. Micro-Raman spectroscopy equipped with a 785 nm laser (DPSSL Driver II, 10 mW) and an MRS-iHR320 modular Raman system was integrated into an Olympus BX53 microscope. A superconducting quantum interference device vibrating sample magnetometer (SQUID, MPMS 3 Quantum Design, USA) was used for measuring the magnetism of Fe_3_O_4_@Chl/Fe nanoparticles.

## Results and discussion

### Synthesis and characterization of Fe_3_O_4_@Chl/Fe CNPs

**Fig. 1:**
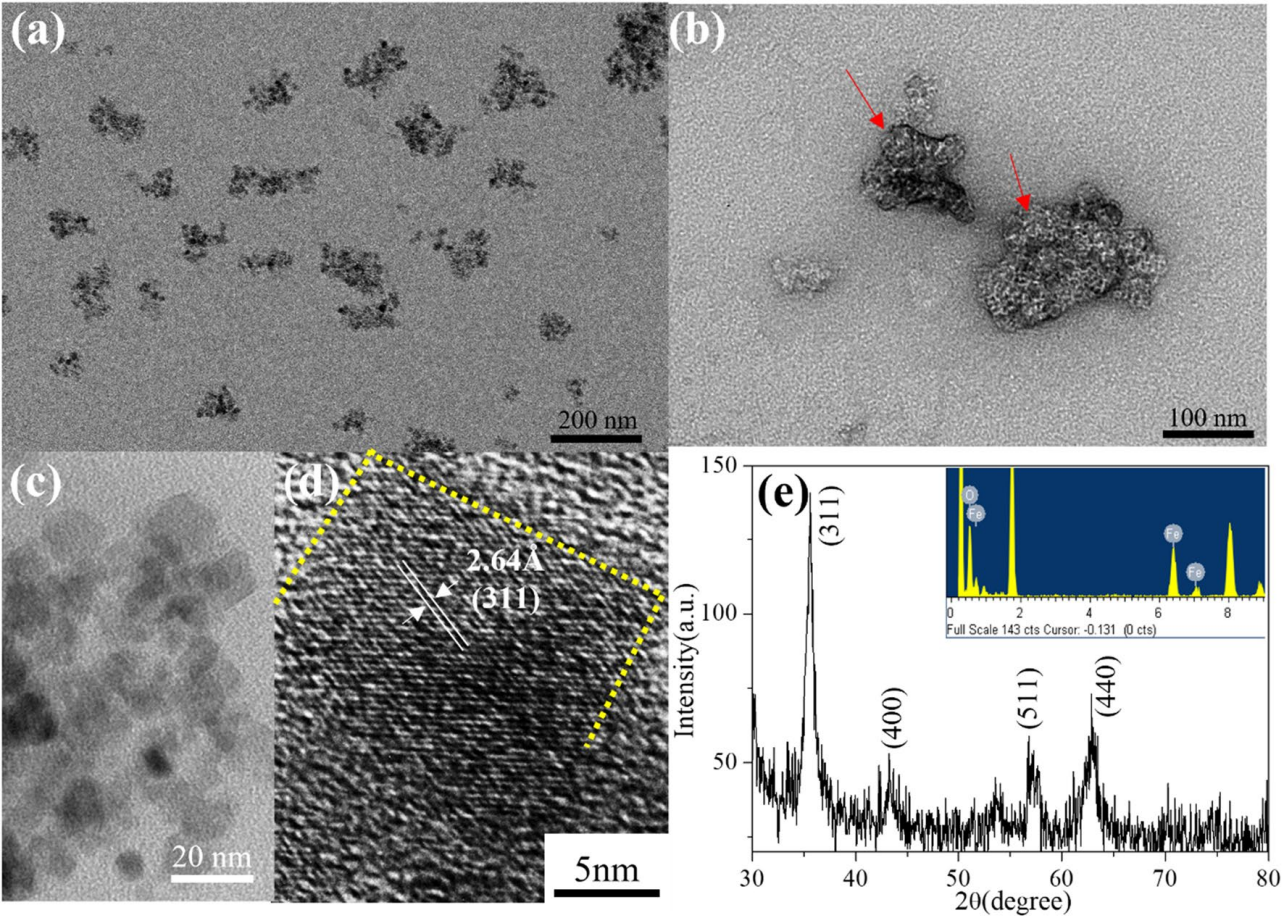
**a** Cryo-EM, **b** negatively stained TEM, **c** high-magnification TEM, and **d** HR-TEM images of Fe_3_O_4_@Chl/Fe CNPs. **e** XRD patterns and EDS analysis (inset) of Fe_3_O_4_@Chl/Fe CNPs. The white dot indicated by the red arrow represents the scattering of inorganic Fe_3_O_4_ embedded in the Chl/Fe matrix under negative staining by uranium tetraacetate

The coprecipitation synthesis method is a popular strategy to fabricate iron oxide nanoparticles. Nevertheless, it has the disadvantage of particle aggregation [38, 39] because the electrostatic and/or steric interactions on the surface of the iron oxide nanocrystals do not offer adequate protection to overcome the magnetic attraction force of each particle. Chl/Fe molecules containing *porphyrin* and carboxylic acid groups possess multiple intermolecular hydrogen bonds and π–π interactions. They are a promising host matrix to modulate the interparticle assembly of iron oxide nanocrystals. Figure 1a shows a cryo-electron microscopy (cryo-EM) image of the Fe_3_O_4_@Chl/Fe CNPs (145.6 ± 31.3 nm) obtained by allowing FeCl_2_, FeCl_3_, HCl, and NH_4_OH to react in the presence of 200 mg of Chl/Fe. Cryo-EM technology is a direct and visual analytic method to determine the real macro/microstructures of Fe_3_O_4_@Chl/Fe CNPs in the liquid phase as cluster-structured particles composed of several primary iron oxide nanocrystals. Figure 1b shows the low electron density within the clustered Fe_3_O_4_@Chl/Fe CNPs using a negative stain, suggesting that these structures were embedded in the Chl/Fe matrix. The high-magnification transmission EM image in Fig. 1c presents several rectangular crystal domains (12.8 ± 4.8 nm) that were closely compacted together. They have a highly ordered lattice fingerprint with a spacing of 0.264 nm, corresponding to the continuous (311) lattice plane of crystalline Fe_3_O_4_ in a single particle (Fig. 1d).

The inset of Fig. 1e shows the presence of Fe, C, and O in the product without other impurities in the EDS measurement. Upon replacing Chl/Fe with Chl-Cu, EDS indicated 9.87% Cu atoms over Cu + Fe and 28.08% N over Fe + Cu + N in the Fe_3_O_4_@Chl/Cu nanocomposites (Additional file 1: Fig. S1). Considering that both Chl/Fe and Chl/Cu derivatives are composed of similar chlorophyll-based structures and act as carboxylate-assisted capping agents, we estimated an ~ 10% fraction of Fe ions from Chl/Fe in the Fe_3_O_4_@Chl/Fe CNPs. As shown in Fig. 1e, the reflection peaks at 35.8°, 43.2°, 57.6°, and 63.2° in the XRD pattern could be assigned to the (311), (400), (511), and (440) planes, respectively, of fcc-structure Fe_3_O_4 _[40, 41]. A strong peak was observed at 670 cm^−1^ in the Raman spectra (Additional file 1: Fig. S2) and was attributed to the A_1g_ mode [40, 41] of Fe_3_O_4_. The lack of the characteristic peaks of Chl/Fe may be due to its low Raman scattering cross-section.

**Fig. 2:**
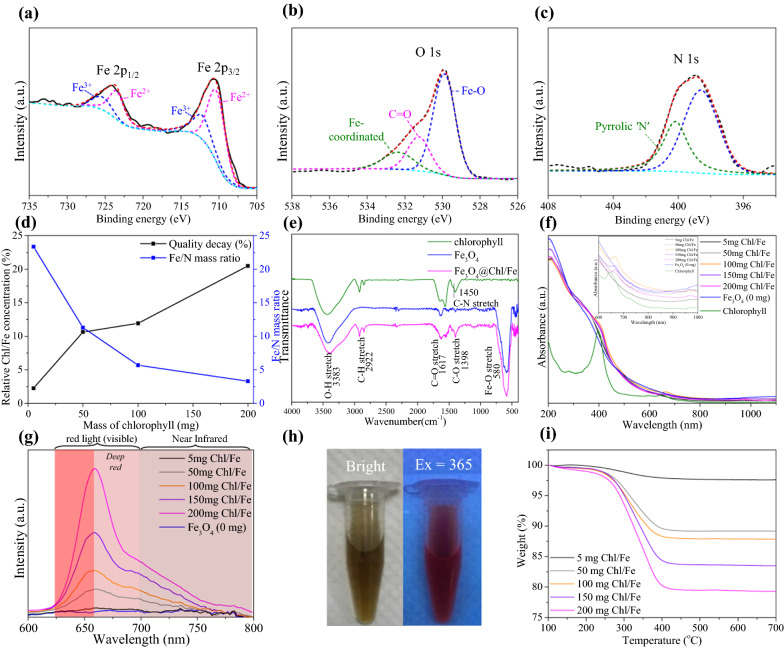
XPS spectra of **a** iron, **b** oxygen, and **c** nitrogen from Fe_3_O_4_@Chl/Fe CNPs. **d** The percentage of Chl weight loss from TGA and Fe/N mass ratios from XPS analysis for different Fe_3_O_4_@Chl/Fe_[0−200 mg]_ CNPs. **e** FTIR absorbance spectra of Chl/Fe, Fe_3_O_4_ and Fe_3_O_4_@Chl/Fe CNPs. **f** UV–Vis spectra of Chl/Fe and Fe_3_O_4_@Chl/Fe_[0−200 mg]_ CNPs. **g** Fluorescence spectrum at 435 nm. **h** Fluorescent image at 365 nm of Fe_3_O_4_@Chl/Fe_[0−200 mg]_ CNPs. **i** TGA measurement of the Fe_3_O_4_@Chl/Fe_[5−200 mg]_ CNPs

Next, we conducted XPS to measure the surface properties of cluster-structured Fe_3_O_4_@Chl/Fe CNPs. As shown in Fig. 2a, the binding energies of Fe 2p_3/2_ and 2p_1/2_ at 710.8 eV and 724.2 eV were determined. The central peak at 529.8 eV was assigned to the O 1s orbital in the crystal structure of the Fe_3_O_4_ material [40], while the additional O 1s peaks at 531.0 and 532.1 eV were related to the Chl capping molecule of Fe_3_O_4_@Chl/Fe CNPs (Fig. 2b) [42]. Figure 2c shows the N 1s peaks at 398.6 and 400.0 eV from two chemically nonequivalent nitrogens, reflecting four central nitrogens and four aza nitrogens [43–45] in the Chl/Fe molecular structure. Accordingly, the Fe/N ratio decreased from 23.2 to 5 mg of Chl/Fe to 3.3 at 200 mg of Chl/Fe based on the XPS measurements (Fig. 2d). In the FTIR spectrum (Fig. 2e), the C–N stretching peak of the Chl/Fe structure at 1450 cm^−1^ was detected for the Fe_3_O_4_@Chl/Fe CNPs [46]. The peaks at 1617 cm^−1^ and 1398 cm^−1^ were attributed to asymmetric and symmetric stretching, respectively, of the COO^−^ functional group due to the coordination of carboxylic acid to the Fe ions [41] on the surface of Fe_3_O_4_ nanocrystals. Furthermore, the peaks at 2856 and 2922 cm^−1^ were attributed to CH_2_ symmetric stretching and asymmetric stretching signals from the Chl/Fe structure. In a control experiment, vibrational peaks from these Chl/Fe-based functional groups were not observed for the bare Fe_3_O_4_ nanocrystals in the Chl/Fe-free synthesis. The Fe–O stretching peak at 580 cm^−1^ was attributed to Fe_3_O_4_@Chl/Fe crystals without Fe_2_O_3_ impurities at 562 cm^−1 ^[47], which was consistent with the Raman results (Additional file 1: Fig. S2).

To understand the assembly of Fe_3_O_4_ nanocrystals by Chl/Fe molecules, 5 mg and 100 mg of Chl/Fe molecules were utilized, and the other synthesis conditions were the same. Additional file 1: Figure S3a shows the increase in the hydrodynamic diameter from 191.9 ± 22.3 nm at 5 mg to 160.8 ± 38.2 nm at 100 mg and 135.9 ± 27.6 nm at 200 mg based on the DLS measurement. In addition to the increase in the overall cluster size with increasing Chl/Fe concentrations, the size distribution of cluster particles became narrower (Additional file 1: Fig. S3b–e). However, Chl/Fe-free Fe_3_O_4_ generated a larger aggregate form (> 233.7 ± 29.8 nm), possibly due to the strong magnetic interaction between the capping-free Fe_3_O_4_ nanocrystals. Figure 2f shows the UV–Vis absorption spectrum of cluster-structured Fe_3_O_4_@Chl/Fe_[0−200 mg]_ CNPs. The bare Fe_3_O_4_ CNPs exhibited a broadened absorption band in the range of 200–1100 nm, which was attributed to ligand-to-metal charge transfer and d–d transitions [40, 41, 48]. Compared with the bare Fe_3_O_4_ CNPs, the Fe_3_O_4_@Chl/Fe CNPs gave rise to an absorption peak at 400–435 nm due to the evolution of the B band transition from the ground state to the second excited state of metal phthalocyanine [49]. At the same time, weak absorption from the Q band of Chl/Fe at 600–700 nm resulted from the transition from the ground state to the first excited state of metal phthalocyanine [50]. Both the B and Q bands became more pronounced when the amount of Chl exceeded 100 mg. Figure 2g shows that the strongest fluorescence intensities at 660 and 700 nm were achieved by high loading of Chl/Fe in the Fe_3_O_4_@Chl/Fe CNPs. The emission areas were 31.1% in the visible region between 625 and 660 nm, as shown in the visual photograph image and observed by the naked eye (Fig. 2h), 43.2% in the deep-red region at 660–700 nm, and 25.5% in the NIR range over 700 nm, making it a promising bioimaging probe to avoid autofluorescence monitoring in the NIR region [51, 52].

Figure 2i shows the thermogravimetric analysis (TGA) results of the cluster-structured Fe_3_O_4_@Chl/Fe_[0−200 mg]_ CNPs prepared using different Chl/Fe concentrations. It can be seen that a slight mass loss of ~ 2% occurred from adsorbed H_2_O below 250 °C for all Fe-based samples. A subsequent residual mass loss of the Chl molecules occurred at 250 °C, and complete decomposition occurred by 435 °C. The ratio of Chl to the Fe_3_O_4_ nanocrystals increased from 2.26% for Fe_3_O_4_@Chl/Fe_[5 mg]_ to 20.5% for Fe_3_O_4_@Chl/Fe_[200 mg]_. Indeed, the increase in Chl-based absorption and fluorescence from the Fe_3_O_4_@Chl/Fe_[200 mg]_ CNPs was due to multilayer adsorption around the Fe_3_O_4_ nanoclusters. In contrast, the magnetization of Fe_3_O_4_ nanocomposites decreased with the reaction at high Chl/Fe concentrations (Additional file 1: Fig. S4), which is related to the decrease in the magnetic Fe_3_O_4_ material population for the abundant Chl/Fe-deposited Fe_3_O_4_@Chl/Fe nanocomposites. In addition, we observed that the positive charge of the Fe_3_O_4_ CNPs (24 mV) was converted to a negative charge at − 44.5 mV upon capping with a dense Chl/Fe organic layer (Fig. 2b, e). This result indicated that the carboxylate groups bound to the iron ions and reoriented the molecular structure through flat stacking to expose the COO^−^ functional groups to water, as illustrated in Scheme 1. Upon replacing the Chl molecule with over 20 mg of citrate ions, amorphous and ultrasmall colloids (Additional file 1: Fig. S5) of the final product were generated (Additional file 1: Fig. S6). In this synthesis lacking π–π conjugation, the tri-carboxylate groups of citrate ions simply coordinate the iron ions, and a strong repulsive force prevents continuous condensation [53, 54].

Several reports have demonstrated the existence of parallel π–π stacking [55–57] in Chl/Fe molecules to guide the self-assembly of nanoparticles through π electrons in the tetrapyrrole ring structure. It is possible that the initial reaction stage of iron and Chl/Fe ions dissolved under acidic conditions triggered the intermolecular self-assembly process of the Chl/Fe-bound Fe species through hydrogen bond formation and π–π stacking. As the NH_4_OH concentration increased, the particle nucleation reaction from the Chl/Fe-bound Fe species progressed, and the COOH groups at the surface of the assembled Chl/Fe-Fe_3_O_4_@Chl/Fe embryo were deprotonated. The subsequent nucleation and growth process under basic conditions prevented the continuous aggregation of primary Fe_3_O_4_ (Additional file 1: Fig. S3b) by the negatively charged repulsion force (Additional file 1: Fig. S3f) of deprotonated Chl/Fe. This improved the dispersion of the cluster-structured Fe_3_O_4_@Chl/Fe CNPs.

**Fig. 3:**
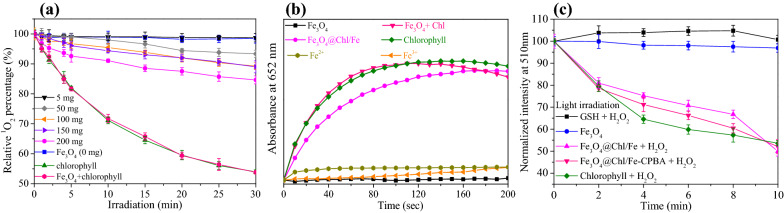
**a** RNO/imidazole assay for the ^1^O_2_ production of Fe_3_O_4_@Chl/Fe_[Chl: 5−200 mg]_ nanoclusters, Fe_3_O_4_ CNPs, Chl/Fe, and the Fe_3_O_4_ CNPs + Chl/Fe mixture as a function of irradiation time with a 660 nm LED light (75 mW/cm^2^). **b** TMB assay for the reaction of iron-based materials at 0.5 ppm_[Fe]_ with 100 mM H_2_O_2_. **c** GSH assay kit for the reactions of the iron-based materials and H_2_O_2_

To prove the potential production of ^1^O_2_ species from the cluster-structured Fe_3_O_4_@Chl/Fe CNPs, we used an RNO/imidazole assay [58] and applied 660 LED light at 75 mW/cm^2^. On the basis of the rate of the change in absorbance at 440 nm in UV–Vis spectra, the particle-mediated production of ^1^O_2_ molecules was observed as a function of irradiation time during 660 nm light exposure (Fig. 3a). A dose-dependent yield of ^1^O_2_ molecules from the Chl/Fe-capped Fe_3_O_4_ nanocrystals was observed, with an 8.16 times higher ^1^O_2_ yield produced by Fe_3_O_4_@Chl/Fe_[200 mg]_ CNPs than by Fe_3_O_4_@Chl/Fe_[5 mg]_ CNPs. The physical mixture of bare Fe_3_O_4_ nanocrystals (100 ppm_[Fe]_) and Chl/Fe (0.2 mM) resulted in almost the same yield as the Chl/Fe solution alone (0.2 mM) (Additional file 1: Fig. S7). Perhaps the quenching effect of the Chl/Fe assemblies in the Fe_3_O_4_@Chl/Fe_[200 mg]_ CNPs originated from partial energy loss via electron or energy transfer from Chl to Fe_3_O_4_ nanocrystals.

As shown in Additional file 1: Fig. S8, the temperature-responsive curves of cluster-structured Fe_3_O_4_@Chl/Fe CNPs at 100 ppm excited by 650 and 808 nm lasers at 75 mW/cm^2^ had T_max_ values lower than 30 °C, and the temperature increases caused by these two laser light energies were not enough to damage cancer cells [59]. Increasing the temperature did not induce apoptosis or cause cell death [60] As the power of the 650 nm laser increased to 210 mW/cm^2^, T_max_ increased to 38.7 °C. The cluster-structured Fe_3_O_4_@Chl/Fe CNPs reached the same temperature after 5 repeated heat/cool cycles with either 650 or 808 nm light at higher laser powers, unlike the photothermally destroyed gold nanoparticles [61].

### Characterization of the Fe_3_O_4_@Chl/Fe catalytic performance and impairment of the redox balance by facilitating GSH oxidation (or degradation) in H_2_O_2_

It has been reported that ferroptosis elicits oxidative stress in CDT cells and preferentially injures cancer cells in the presence of a high H_2_O_2_ concentration [13, 16]. To clarify the CDT functionalities of the Fe_3_O_4_@Chl/Fe CNPs, a TMB assay was applied to detect the hydroxyl radicals produced from H_2_O_2_ catalysis by Fe-based CNPs. The parameters of the 0.5 ppm_[Fe]_ Fe-based samples and 100 mM H_2_O_2_ concentration are adopted in the following examination of ROS generation (Fig. 3b) because their signal change rates are rapid and can be clearly distinguished when compared with the same reaction at 1 mM H_2_O_2_ (Additional file 1: Fig. S9b). H_2_O_2_ (50–1000 mM) is commonly used to access peroxidase-like activity at 652 nm in the TMB assay [62–64]. Due to their low optical density at 652 nm, the separation of the Fe_3_O_4_@Chl/Fe CNPs is negligible in the TMB assay measurement (Additional file 1: Fig. S9c). Compared with pure Chl/Fe, which loses its catalytic activity quickly after reaching its maximum, cluster-structured Fe_3_O_4_@Chl/Fe CNPs exhibited preferable steady-state kinetics despite rapid conversion time. The conversion rate from TMB to oxTMB was 5.1% by Fe_3_O_4_ alone, 75.0% by Chl/Fe, and 72.5% by cluster-structured Fe_3_O_4_@Chl/Fe CNPs, as determined from the extinction coefficient of 39,000 M^−1^ cm^−1^ for TMB at 652 nm. Inactive nanozyme properties were observed for Fe_3_O_4_ alone. In contrast, the Chl/Fe assemblies in the Fe_3_O_4_@Chl/Fe_[200 mg]_ CNPs demonstrated a remarkable improvement in peroxidase activity. Clearly, the exposure of Chl/Fe species at the Fe_3_O_4_@Chl/Fe_[200 mg]_ CNPs could facilitate the interaction with H_2_O_2_ and improve the peroxidase-like active sites of Fe_3_O_4_@Chl/Fe CNPs.

Furthermore, we found that the rate of TMB oxidation decreased upon the addition of GSH (0.1 mM), a typical bioantioxidant within cells, in the cluster-structured Fe_3_O_4_@Chl/Fe CNPs and H_2_O_2_ solution (Additional file 1: Fig. S10). Another experiment with a GSH detection kit showed the consumption of GSH by reaction with cluster-structured Fe_3_O_4_@Chl/Fe CNPs and H_2_O_2_ (Additional file 1: Fig. S11). This result suggested the competitive oxidation reactions of GSH → GSSG and TMB → oxTMB through the CDT process involving the peroxidase activity of cluster-structured Fe_3_O_4_@Chl/Fe CNPs to potentially modulate the antioxidation microenvironment of cancer cells. Intriguingly, we observed enhanced depletion of GSH when the oxidation effects of the OH radical from the catalytic H_2_O_2_ and ^1^O_2_ species from the PDT were included (Fig. 3c and Additional file 1: Fig. S12). Such a decrease in the GSH concentration is superior to those observed under PDT or CDT alone, indicating that the combination therapy may be a mutually beneficial method to treat cancer cells by inducing intracellular ROS production, increasing the ferroptosis effect by PDT, and prolonging the therapeutic duration.

### CPBA-modified Fe_3_O_4_@Chl/Fe nanoparticles induce cytotoxicity and enhance BC cell PDT efficacy

**Fig. 4:**
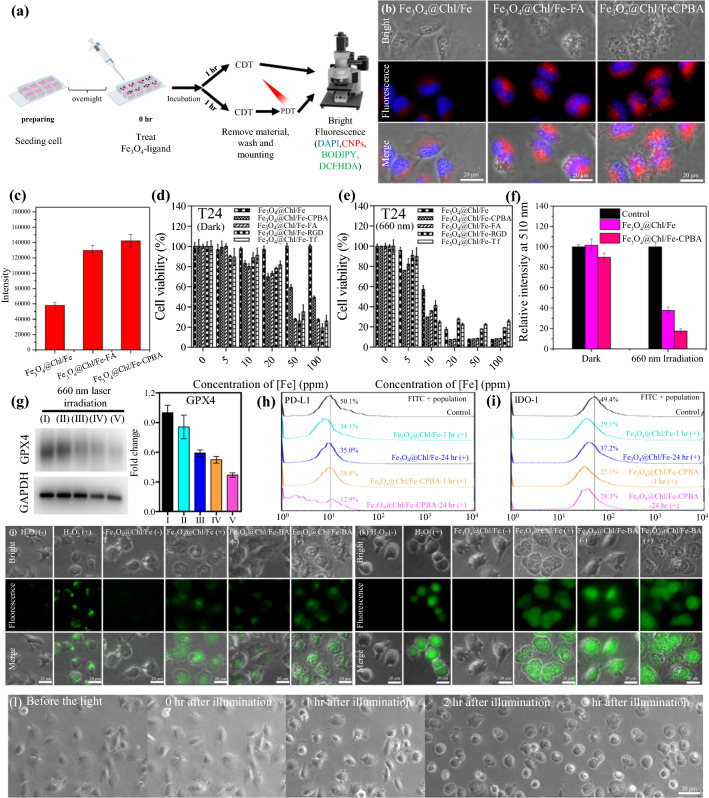
**a** Schematic illustrations of T24 cells that received CDT and PDT by incubation with CPBA-modified Fe_3_O_4_@Chl/Fe CNPs. **b** Bright-field and fluorescence microscopy images of T24 cells incubated with ligand-free, CPBA-modified, and FA-modified Fe_3_O_4_@Chl/Fe CNPs. Blue: DAPI; red: Chl/Fe fluorescence. **c** The corresponding quantification of cellular fluorescence imaging from the samples in **b**. MTT assays for the **d** dark and **e** 660 nm-light reactions of T24 cells with different sample treatments. **f** Intracellular GSH of T24 cells upon treatment with Fe_3_O_4_@Chl/Fe and Fe_3_O_4_@Chl/Fe-CPBA CNPs for 24 h with dark or 660 nm-irradiated treatments. **g** Western blot analysis of GPX4 expression levels and flow cytometry assays of **h** PD-L1 and **i** intracellular IDO-1 expression in T24 cells after treatment with 20 ppm_[Fe]_ Fe_3_O_4_@Chl/Fe CNPs and 20 ppm_[Fe]_ CPBA-modified Fe_3_O_4_@Chl/Fe CNPs. (I) Control, (II) Fe_3_O_4_@Chl/Fe-1 h (+), (III) Fe_3_O_4_@Chl/Fe-24 h (+), (IV) Fe_3_O_4_@Chl/Fe-CPBA-1 h (+), (V) Fe_3_O_4_@Chl/Fe-CPBA-24 h. Bar graphs were constructed according to the GPX4 protein expression level normalized to GAPDH. **j** BODIPY 581/591-C11-stained and **k** DCFH-DA-stained T24 cells incubated with 1 mM H_2_O_2_, 20 ppm_[Fe]_ Fe_3_O_4_@Chl/Fe CNPs and 20 ppm_[Fe]_ CPBA-modified Fe_3_O_4_@Chl/Fe CNPs for 1 h. The (+)-labeled groups indicate the light-treated reaction at 20 ppm iron and a laser power density of 75 mW/cm^2^ for 1 min after 60 min of coculture and further incubation for another 30 min. **l** Apoptosis- and ferroptosis-induced cell shrinkage is identified by the change in cell morphology after treatment with 20 ppm_[Fe]_ CPBA-modified Fe_3_O_4_@Chl/Fe CNPs with a 660 nm laser over time

Before PDT examinations, the toxicity effect of Fe_3_O_4_@Chl/Fe CNPs on T24 cells was evaluated by the MTT assay, which showed 98% cell viability after 24 h of coculture (Fig. 4a). Furthermore, Fe_3_O_4_@Chl/Fe CNPs were modified with CPBA molecules to improve targeted delivery via biorecognition of glycoprotein receptors [8], which are highly expressed in BC cells and other cancer cell types [8]. AAS measurements determined the rapid accumulation of Fe_3_O_4_@Chl/Fe-CPBA CNPs onto/into T24 cells at 1 h, which reached a plateau between 16 and 24 h of treatment (Additional file 1: Fig. S13). Figure 4b presents a fluorescence microscopy image of the brightened red fluorescent signal within T24 cells after 24 h of treatment with Fe_3_O_4_@Chl/Fe-CPBA CNPs. The corresponding fluorescence intensity was 2.3 times higher than that of the CPBA-free Fe_3_O_4_@Chl/Fe CNP group (Fig. 4c and Additional file 1: Fig. S13). These Fe_3_O_4_@Chl/Fe-CPBA CNPs were internalized into T24 cells and visualized by confocal laser scanning microscopy (CLSM), as shown in Additional file 1: Fig. S15. In contrast to the low cytotoxicity of Fe_3_O_4_@Chl/Fe CNPs, the CPBA-modified Fe_3_O_4_@Chl/Fe_[200 mg]_ CNPs exhibited a dose-dependent harmful effect on malignant T24 cells (Fig. 4e). A similar cell death tendency was observed after treatment with Fe_3_O_4_@Chl/Fe CNPs modified with transferrin (Tf, to target TF receptors), arginylglycylaspartic acid (RGD, to target integrin receptors), and folic acid (FA to target folate receptors) (Fig. 4d, e). Figure 4b, c shows that FA-modified Fe_3_O_4_@Chl/Fe_[200 mg]_ CNPs, for example, were also efficiently internalized into cancer cells. These CNPs did not induce significant toxicity to normal cells, including SV-HUC1, 3T3, and VERO cells (Additional file 1: Figs. S15a, b, S16c–f). They enabled HeLa cervical cancer (Additional file 1: Fig. S16a, b) and mouse bladder carcinoma MB49 (Additional file 1: Fig. S17a, b) cell death. Reasonably, the targeted delivery would enhance the endocytosis of Fe_3_O_4_@Chl/Fe CNPs for subsequent interaction with cancerous H_2_O_2_ to induce cell death via CDT.

Upon PDT treatment of T24 cells with CPBA-modifiedFe_3_O_4_@Chl/Fe_[200 mg]_ CNPs at 660 nm (75 mW/cm^2^), almost twofold cell viability was observed when Fe_3_O_4_@Chl/Fe_[200 mg]_ CNPs were applied at 10–20 ppm_[Fe]_ (Fig. 4d, e), and the Fe_3_O_4_@Chl/Fe_[5 mg]_ CNPs had negligible PDT-induced phototoxicity (< 10%) at 10–50 ppm_[Fe]_ (Additional file 1: Fig. S18a). Again, we observed a similar cell death profile (Additional file 1: Fig. S17) for mouse bladder carcinoma MB49 cells after efficient delivery of CPBA-modified Fe_3_O_4_@Chl/Fe_[200 mg]_ CNPs (Additional file 1: Fig. S19). It is worth mentioning the absence of a photothermal effect from Fe_3_O_4_@Chl/Fe_[200 mg]_ CNPs during PDT treatment at 75 mW/cm^2^ (Additional file 1: Fig. S8).

### ROS-induced lipid peroxidation by Fe_3_O_4_@Chl/Fe-CPBA CNPs

We further investigated whether the targeted delivery of Fe_3_O_4_@Chl//Fe-CPBA CNPs reinforced intracellular ROS and lipid peroxidation levels in T24 cells. As shown in Fig. 4j, Fe_3_O_4_@Chl//Fe-CPBA CNPs exhibited moderate fluorescence in BODIPY (LPO)-stained cells, indicating slight lipid peroxidation before PDT, and the cells that received Fe_3_O_4_@Chl/Fe CNPs alone merely exhibited decreased expression of LPO. Notably, there was no significant difference in the depletion of antioxidation species between Fe_3_O_4_@Chl/Fe CNPs and Fe_3_O_4_@Chl/Fe-CPBA CNPs according to the GSH level for aging in the dark (Additional file 1: Fig. S11) and PDT groups (Fig. 3c and Additional file 1: Fig. S12). However, the corresponding green fluorescence of LPO-stained cells by the CPBA-modified particle-treated T24 cells plus 660 light generated strong emission intensity, indicating the increased LPO from the CDT process after 0.5 h of PDT. Indeed, Fe_3_O_4_@Chl/Fe-CPBA CNPs exhibited pharmacological inhibition to decrease GPX4 protein levels, sensitizing cancer cells to ferroptosis after PDT (Fig. 4g). Furthermore, Fe_3_O_4_@Chl/Fe-CPBA CNPs showed the potential to inhibit the expression of immunosuppressive factors, including PD-L1 and IDO-1 (Fig. 4h–i). IDO-1 recognizes an enzyme involved in tryptophan synthesis, which may cause the deprivation of tryptophan, an essential amino acid for cytotoxic T cells [65]. At the same time, the intracellular ROS levels also increased, as observed in the DCFH-DA-stained cells (Fig. 4k), which was consistent with the initiation of ROS production by the Fe_3_O_4_@Chl/Fe CNPs at 0.1-1 mM H_2_O_2_ of the intracellular environment in cancer cells [13, 16] in the aforementioned TMB and GSH depletion assays (Additional file 1: Figs. S9b and S14). Figure 4L shows that the cells that underwent LPO-assisted PDT exhibited significant shrinkage of the cellular structure at 2 h posttreatment, similar to the pattern of apoptosis. With less internalization of Fe_3_O_4_@Chl/Fe CNPs into cancer cells, there was a lack of obvious ROS and LPO responses of cells. Thus, we proposed that the depletion of GSH (Fig. 4f) by ferroptosis induced by Fe_3_O_4_@Chl/Fe@CPBA CNPs in the initial incubation period of treatment would decrease the antioxidation capability for the subsequent multiplex cooperation effects: (i) considerable induction of ferroptosis cell death by depleting GSH after PDT, (ii) impaired redox balancing, (iii) weakened antioxidation and tumor progression capacity for later injury via continued exposure to oxygen species of CDT and PDT, and (iv) revitalized PDT to kill cells with lower GSH levels by locally intensified photooxidation.

### Combining CDT–PDT in an orthotopic BC mouse model

**Fig. 5:**
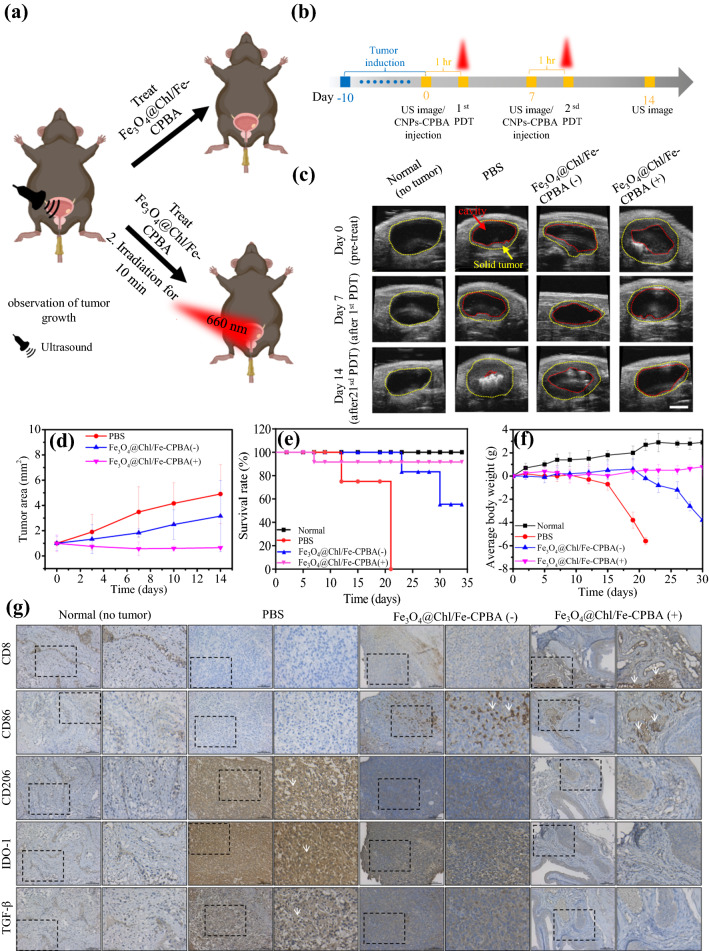
In vivo therapeutic efficacy assessment of Fe_3_O_4_@Chl/Fe-CPBA CNPs with CDT and CDT–PDT treatments. **a** Scheme for the treatment of tumor induction, local sample loading (1 h), and laser-triggered PDT reactions for BC in vivo with US imaging monitoring. **b** Flow chart for the timeline of nanoparticle-mediated CDT and PDT experiments in vivo. **c** US images of orthotopic bladder tumors at different time points after treatment (scale bar: 2 mm). **d** The tumor growth curves calculated from the US images in **c**. **e** The survival curves of different treatment groups. **f** The body weight changes of different treatment groups. **g** IHC staining results of CD8, PD-L1, IDO-1, CD206, CD86, and TGF-β in the different treatment groups. The (+)-labeled groups indicate the light-treated reaction at a laser power density of 75 mW/cm^2^ for 10 min. Scale bar = 100 μm

To prove the CDT-boosted antitumor efficacy of the PDT concept, we used a mouse orthotropic MB49-bearing model with noninvasive US monitoring of tumor growth in a timely manner (Fig. 5a). All the tumor-bearing mice were randomly grouped into four groups (PBS, only intrabladder administration of PBS; Fe_3_O_4_@Chl/Fe-CPBA(−), intrabladder administration of 100 ppm Fe_3_O_4_@Chl/Fe-CPBA CNPs for 1 h without laser irradiation; Fe_3_O_4_@Chl/Fe-CPBA(+), intrabladder administration of Fe_3_O_4_@Chl/Fe-CPBA CNPs for 1 h followed by 10 min of 660 nm laser irradiation and two light dose treatments with one-week intervals) (Fig. 5b). To analyze the US image results of tumor-implanted mice, the contrast difference between the yellow dotted circle of the original size of the bladder and the red dotted circle from the size of the bladder cavity remaining after the cancer cells was accounted for (Fig. 5c). The group with a BC area of ~ 10 mm^2^ after 10 days was included (Fig. 5b), defined as Day 0. Regarding the size of the bladder tumor that was subjected to treatments, the area calculated is shown in the yellow dotted circle, followed by normalization to the size on Day 0, which is represented as the fold change (Fig. 5d). In contrast to the smooth normal bladder wall, the inoculated tumor cells were attached to the bladder wall and formed irregular deformations (red dotted circle) (Fig. 5c). US imaging of the bladder space in the mice treated with PBS showed remarkable tumor growth around the bladder wall with a thickness of 2.4 mm at Day 7 and almost complete occupation by the tumor mass at Day 14 (Fig. 5c). However, the Fe_3_O_4_@Chl/Fe-CPBA(−)-treated mice had retarded tumor growth (Fig. 5c, d, blue line). This result suggested that Fe_3_O_4_@Chl/Fe-CPBA nanocomposites (such as CDT) may trigger ferroptosis and thus suppress the progression of the tumor (Fig. 5a). Under this ferroptosis condition, more than 50% of the mice had a 60% extension of the survival time to over 5 weeks, while mice treated with only PBS solution died after 3 weeks (Fig. 5e). US imaging revealed almost complete tumor remission (Fig. 5c, d, purple line) in the Fe_3_O_4_@Chl/Fe-CPBA(+)-treated group. This result indicated that CDT-promoted PDT efficacy was associated with Fe_3_O_4_@Chl/Fe-CPBA CNP-mediated photolysis of the bladder tumor. Furthermore, the resulting bladder tumor-bearing mice achieved a 91.7% survival rate even after 5 weeks of photolysis.

**Fig. 6:**
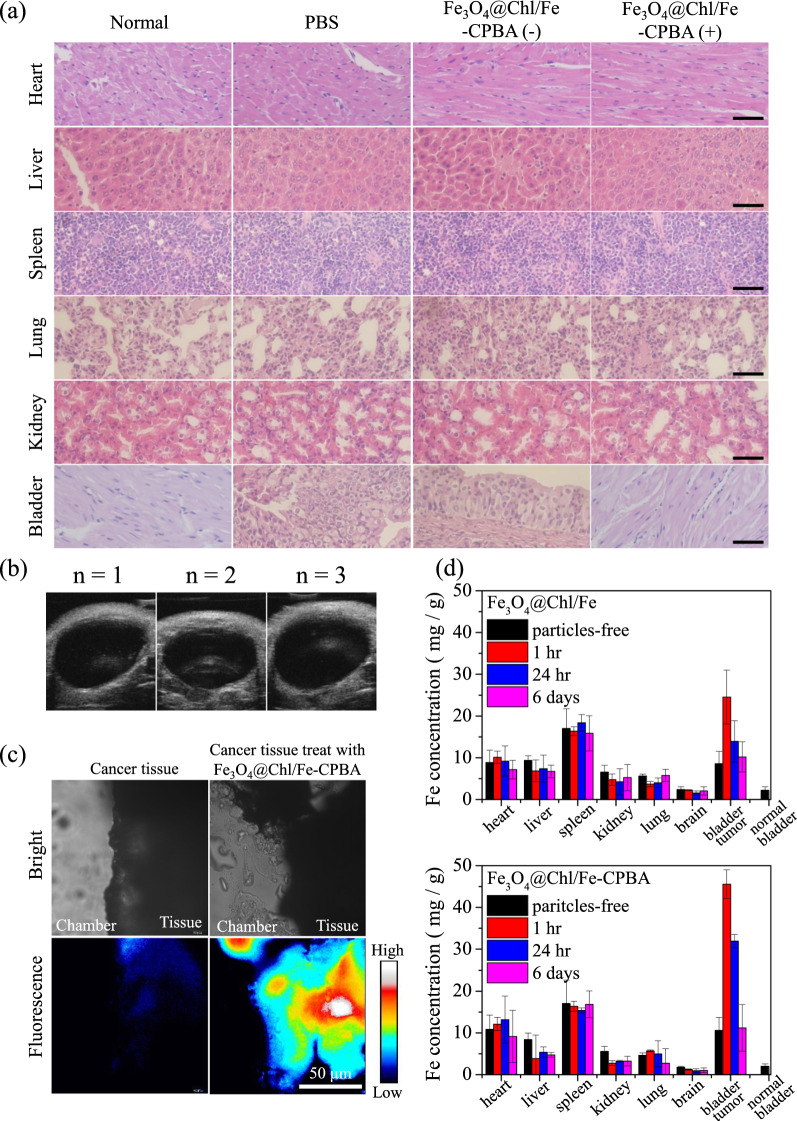
**a** H&E-stained tissue sections of major organs (heart, liver, spleen, lungs, kidneys, and bladders) isolated from mice after different treatments. Scale bar: 50 μm. **b** US images of the mouse bladder after 3 months of Fe_3_O_4_@Chl/Fe-CPBA(+) treatment. **c** Fluorescence image of the mouse bladder treated with Fe_3_O_4_@Chl/Fe-CPBA for 1 h. Scale bar: 50 μm. **d** Organ biodistribution studies of Fe_3_O_4_@Chl/Fe-CPBA CNPs in major organs following bladder administration. Organ biodistribution profiles at a variety of times post-injection

Body weight also reflected the therapeutic efficacy in different treatment groups (Fig. 5f). The mice in the PBS treatment group showed marked body weight loss after Day 14, which might be due to occupation of the bladder space by the large tumor mass, which blocks urine excretion and finally results in kidney failure and death. Similar trends were observed in the Fe_3_O_4_@Chl/Fe-CPBA CNP-treated mice, but the timing of the decrease in the body weight curve was delayed to Day 23, which revealed that induced ferroptosis could only delay the progression of the tumor but not cure cancer. Only mice that underwent Fe_3_O_4_@Chl/Fe-CPBA CNPs and PDT treatment had no body weight reduction throughout the experimental period (Fig. 5f). Pathology evaluation of mouse organs was prepared, stained with H&E, and imaged to evaluate the possible systemic adverse effects of Fe_3_O_4_@Chl/Fe-CPBA CNPs (Fig. 6a). There were no noticeable cell morphology changes in the mouse bladder after the CDT-elicited PDT process compared to healthy bladder tissue. Compared to the PBS control group, there was no apparent damage or inflammation in the heart, liver, spleen, lung, or kidney of Fe_3_O_4_@Chl/Fe-CPBA-treated mice. These results demonstrated the lack of general toxicity of Fe_3_O_4_@Chl/Fe-CPBA CNPs in mouse models and verified local ferroptosis via intravesical instillation combined with photodynamic therapy.

An immunostimulation effect was also found in the Fe_3_O_4_@Chl/Fe-CPBA CNP-treated group. As illustrated in Fig. 5g, immunosuppressive markers such as PD-L1, IDO-1, and TGF-β in the tumor section were markedly decreased in the Fe_3_O_4_@Chl/Fe-CPBA(−) CNP- and Fe_3_O_4_@Chl/Fe-CPBA(+) CNP-treated groups. On the contrary, the expression of CD8 was increased in the tumor section of Fe_3_O_4_@Chl/Fe-CPBA(−) CNP- and Fe_3_O_4_@Chl/Fe-CPBA(+) CNP-treated groups. Although no apparent tumor remained in the Fe_3_O_4_@Chl/Fe-CPBA(+) CNP group, we used the injection area of bladder tissue to identify these markers. Furthermore, the significant population of infiltrated macrophages in tumor tissue was also represented as an inflammatory M1-like phenotype (CD86^+^) instead of a tumor-associated M2-like phenotype (CD206^+^). These results indicated that Fe_3_O_4_@Chl/Fe-CPBA(+) CNP treatment shows the potential to reprogram the BC tumor microenvironment from cold (immune depletion) to hot (immune activation).

Figure 6b shows the follow-up US imaging observations of the mouse bladder after CDT–PDT treatment. The bladder cavity completely recovered and appeared empty without recurrence within three months. However, Fe_3_O_4_@Chl/Fe-CPBA CNPs could bind to the cancer cells at the tumor surface area (Fig. 6c) after 1 h of incubation. AAS measurement (Fig. 6d) of the biodistribution result showed an enhanced accumulation of Fe_3_O_4_@Chl/Fe-CPBA CNPs at the tumor site within the bladder at 1 h in comparison with the particle-free group (bladder cancer-bearing mice without treatment) and the Fe_3_O_4_@Chl/Fe CNPs in the bladder tumor at 1 h. The lower Fe concentration in the normal bladder (without tumor) compared to the heart, liver, and bladder with tumor tissue was consistent with previously reported findings [66]. Observing the Fe ions in the bladder from 1 h to 24 has been decreased by 31.1% in Fe_3_O_4_@Chl/Fe-CPBA CNPs but by 45.8% in Fe_3_O_4_@Chl/Fe CNPs. It was demonstrated that the Fe_3_O_4_@Chl/Fe-CPBA CNPs could prolong retention in the bladder tumor site through specific targeting. Although they were indeed excreted from the bladder from Day 1 to Day 6 via the flushing by mouse over time, certain residual Fe-based materials appear in the bladder which may require a longer time to metabolize after treatment. Fortunately, we did not find an increase in Fe ions in the other organs on 6 days after treatments, indicating that these CNPs did not enter systemic circulation. Based on CDT-elicited PDT reactions, this developed catheter-guided intrabladder administration method for Fe_3_O_4_@Chl/Fe-CPBA CNPs serves as a new therapeutic strategy to reduce the antioxidation effect of the tumor, to improve the PDT efficiency and to prevent the uncertain toxicity of long-term drug accumulation in the bladder.

## Conclusion

In summary, this study successfully demonstrated the green facile synthesis of Fe_3_O_4_@Chl/Fe CNPs with CDT-enhanced PDT function for BC treatment by regulating the redox balance and reprogramming the TME. The high density of Chl/Fe around Fe_3_O_4_ resulted in high dispersion and fluorescence performance of the Fe_3_O_4_@Chl/Fe CNPs. The modification of Fe_3_O_4_@Chl/Fe CNPs by CPBA may target glycoproteins on BC cells and thus vastly enhance the internalization of Fe_3_O_4_@Chl/Fe CNPs into BC cells. The internalized Fe-based nano-photosensitizers initially induced the CDT-mediated Fenton reaction to deplete GSH and downregulate GPX4; on the other hand, they may also boost lipid peroxidation-mediated ferroptosis in combination with PDT. Our CDT–PDT therapy greatly inhibited tumor growth, increased the survival rate of orthotopic MB49-bearing mice from 0 to 91.7%, and induced immunostimulatory-related markers. Additionally, our results proved that CDT–PDT may reverse and remodel the TIME into an immunostimulatory microenvironment through PD-L1 inactivation. Immunosuppressive factor expression and M2-like macrophage accumulation suppressed by CDT–PDT therapy may help to alleviate tumor relapse located outside the irradiated area. Localized administration and restricted PDT regions prevented the systemic circulation of nanomaterials and minimized potential toxicity, as proven by the lack of significant body weight loss or significant organ damage after treatment in orthotopic MB49-bearing mice. This study offers ideas about CDT-mediated ferroptosis-enhanced PDT nano-immunotherapeutics fabricated through novel material design and synthesis.

## Supplementary Information

**Additional file 1:Figure S1.** EDS measurement of the elemental composition ofthe Fe_3_O_4_@Chl/Cu CNPs. **Figure S2.** Raman spectra of the Fe_3_O_4_@Chl/FeCNPs synthesized with different Chl/Fe concentrations, capping-free Fe_3_O_4_nanocrystals, and Chl/Fe molecules. **Figure S3.** (a) DLS, (b) zeta potential, and (c–f) the sizedistribution of cluster particles of Fe_3_O_4_@Chl/Fe CNPssynthesized with different Chl/Fe concentrations. **Figure S4.** SQUID measurement of Fe_3_O_4_@Chl/Fe_p[0–200 mg]_ CNPs at 300 K. **Figure S5.** (a) Photographs and (b–e) TEM images of Fe_3_O_4_nanocrystal solutions with different citric acid concentrations. TEM images:(b) 2.1 mg (c) 10.6 mg (d) 21.2 mg (e) 42.4 mg. **Figure S6.** X-ray diffraction pattern of Fe_3_O_4_nanocrystals synthesized by using different citrate acid concentrations. **Figure S7.** RNO/imidazole assay for theproduction of singlet oxygen species at 25 ppm_[Fe]_ from the Fe_3_O_4_@Chl/FeCNPs, Fe_3_O_4_, Chl/Fe, and physically mixed Fe_3_O_4_+Chl/Femolecules upon 660 nm light irradiation for 10 min. **Figure S8.** Thermal cycle curves of the Fe_3_O_4_@Chl/Fe_p[200 mg]_ CNPs ([Fe] = 100 ppm) under (a) 650 nm and (b) 808 nm laserirradiation. (c) Thermal cycle curves of Chl/Fe ([Chl/Fe] = 0.2 mM) under 650 nm and 808 nm laser irradiation. **Figure S9.** In vitro characterizations of the chromogenic performance of a varietyof Fe_3_O_4_@Chl/Fe_[0–200 mg]_ CNPs ([Fe] = 0.5 ppmfrom AAS and [Chl/Cu] = 0.001 mM from UV–Visible measurements). Fe_3_O_4_–N_2_H_4_is the iron oxide from reference 45. (b) TMB assay for the reaction of iron-basedmaterials at 0.5 ppm_[Fe]_ with 1 mM H_2_O_2_. (c) UV–Vis spectra of Fe_3_O_4_@Chl/Fe_[200 mg]_ CNPs at 0.5 ppm_[Fe]_. **Figure S10.** TMB assay for evaluating the reaction of H_2_O_2_with (a) 0.5 ppm_[Fe]_ Fe_3_O_4_@Chl/Fe_[200 mg]_CNPs and 0.5 ppm_[Fe]_ ppm_[Fe]_ Fe_3_O_4_@Chl/Fe_[200 mg]_ CNPs plus 1 mM GSH and (b) 0.5 ppm_[Fe]_ Chl/Fe molecule andChl/Fe molecule plus 1 mM GSH. **Figure S11.** GSH depletion assay under (a) 20 ppm_[Fe]_Fe-based treatments and (b) 20 ppm_[Fe]_ Fe-based treatments combinedwith a 100 μM H_2_O_2_ solution. **Figure S12.** GSH depletion assay with 20 ppm Fe_3_O_4_@Chl/Fe_[200 mg]_ CNPs and Fe_3_O_4_@Chl/Fe_[200 mg]_-CPBACNPs combined with irradiation by a 75 mW/cm^2^ 660 nm laser. **Figure S13.** AAS measurements for theuptake analysis of (a) T24 cancer cells, (b) MB49 cancer cells, (c) SV-HUC1normal cells, and (d) Vero normal cells after treatment with Fe_3_O_4_@Chl/Fe_[200 mg]_ and Fe_3_O_4_@Chl/Fe_[200 mg]_-CPBA CNPsfor 1, 16 and 24 h. **Figure S14.**Confocal images of T24 cells treated with Fe_3_O_4_@Chl/Fe_[200 mg]_-CPBA CNPs. (a) Fluorescence and confocal images from bottom to top (b–d).(scale bar: 20 μm). **Figure S15.** Cellviability after treated with different concentrations of modified Fe_3_O_4_@Chl/Fe_[200 mg]_ CNPs with targeting molecules such as CPBA, FA, RGD, and transferrin(a) without and (b) with a 660 nm LED light source are displayed. **Figure S16.** The cell viability of HeLa,NIH 3T3 and VERO cells treated with Fe_3_O_4_@Chl/Fe_[200 mg]_ CNPs group and CPBA-, RGD-, andtransferrin-conjugated Fe_3_O_4­_@Chl/Fe_[200 mg]_ CNPs without (a, c, e) and with (b, d, f) a 660 nmLED light source at 75 mW/cm^2^. **Figure S17.** The cell viability of MB49 treated with Fe_3_O_4_@Chl/FeCNPs and Fe_3_O_4_@Chl/Fe-CPBA CNPs (a) without and (b) withirradiation by a 660 nm LED light source at 75 mW/cm^2^. **Figure S18.**The viability of T24 cells treated with Fe_3_O_4_@Chl/Fe_[5 mg and 200 mg]_ CNPs synthesized with 5 and 200 mg of Chl/Fe for 24 h. (a)Without light exposure (b) exposed to 660 nm LED light (75 mW/cm^2^)([Fe] = 0.2, 1, 5, 10, 50, 100 ppm). **Figure S19.** Confocal images of MB49 cells treated with Fe_3_O_4_@Chl/Fe_[200 mg]_-CPBA CNPs after24 h: (a) Fluorescence and confocal images from bottom to top (b–d). Scale bar:20 μm.

## Data Availability

Not applicable.
