# Supplementary material for: Iron oxide@chlorophyll clustered nanoparticles eliminate bladder cancer by photodynamic immunotherapy-initiated ferroptosis and immunostimulation
Source: J Nanobiotechnology. 2022 Aug 11;20:373. doi: 10.1186/s12951-022-01575-7 (PMC9367122; doi:10.1186/s12951-022-01575-7)
Supplement: Supplementary file 1 — Additional file 1:Figure S1. EDS measurement of the elemental composition ofthe Fe3O4@Chl/Cu CNPs. Figure S2. Raman spectra of the Fe3O4@Chl/FeCNPs synthesized with different Chl/Fe concentrations, capping-free Fe3O4nanocrystals, and Chl/Fe molecules. Figure S3. (a) DLS, (b) zeta potential, and (c–f) the sizedistribution of cluster particles of Fe3O4@Chl/Fe CNPssynthesized with different Chl/Fe concentrations. Figure S4. SQUID measurement of Fe3O4@Chl/Fep[0–200 mg] CNPs at 300 K. Figure S5. (a) Photographs and (b–e) TEM images of Fe3O4nanocrystal solutions with different citric acid concentrations. TEM images:(b) 2.1 mg (c) 10.6 mg (d) 21.2 mg (e) 42.4 mg. Figure S6. X-ray diffraction pattern of Fe3O4nanocrystals synthesized by using different citrate acid concentrations. Figure S7. RNO/imidazole assay for theproduction of singlet oxygen species at 25 ppm[Fe] from the Fe3O4@Chl/FeCNPs, Fe3O4, Chl/Fe, and physically mixed Fe3O4+Chl/Femolecules upon 660 nm light irradiation for 10 min. Figure S8. Thermal cycle curves of the Fe3O4@Chl/Fep[200 mg] CNPs ([Fe] = 100 ppm) under (a) 650 nm and (b) 808 nm laserirradiation. (c) Thermal cycle curves of Chl/Fe ([Chl/Fe] = 0.2 mM) under 650 nm and 808 nm laser irradiation. Figure S9. In vitro characterizations of the chromogenic performance of a varietyof Fe3O4@Chl/Fe[0–200 mg] CNPs ([Fe] = 0.5 ppmfrom AAS and [Chl/Cu] = 0.001 mM from UV–Visible measurements). Fe3O4–N2H4is the iron oxide from reference 45. (b) TMB assay for the reaction of iron-basedmaterials at 0.5 ppm[Fe] with 1 mM H2O2. (c) UV–Vis spectra of Fe3O4@Chl/Fe[200 mg] CNPs at 0.5 ppm[Fe]. Figure S10. TMB assay for evaluating the reaction of H2O2with (a) 0.5 ppm[Fe] Fe3O4@Chl/Fe[200 mg]CNPs and 0.5 ppm[Fe] ppm[Fe] Fe3O4@Chl/Fe[200 mg] CNPs plus 1 mM GSH and (b) 0.5 ppm[Fe] Chl/Fe molecule andChl/Fe molecule plus 1 mM GSH. Figure S11. GSH depletion assay under (a) 20 ppm[Fe]Fe-based treatments and (b) 20 ppm[Fe] Fe-based treatments combinedwith a 100 μM H2O2 solution [file 12951_2022_1575_MOESM1_ESM.docx]

**Supporting Information**

**Iron oxide@chlorophyll clustered nanoparticles eliminate bladder cancer by photodynamic immunotherapy-initiated ferroptosis and immunostimulation.**

Yu-Cheng Chin,^a^ Li-Xing Yang,^a^ Fei-Ting Hsu,^b*^ Che-Wei Hsu,^c^ Te-Wei Chang,^d^ Hsi-Ying Chen,^e^ Linda Yen-Chien Chen,^f^ Zi Chun Chia,^a^ Chun-Hua Hung,^g^ Wu-Chou Su,^g^ Yi-Chun Chiu_,_^d,h,i*^ Chih-Chia Huang_,_^a,g,j*^ and Mei-Yi Liao^e*^

1. Department of Photonics, National Cheng Kung University, Tainan 70101, Taiwan.
2. Department of Biological Science and Technology, China Medical University, Taichung 406, Taiwan.
3. Division of Urology, Department of Surgery, Taipei City Hospital Zhongxing Branch, Taipei 103, Taiwan
4. Division of Urology, Department of Surgery, Taipei City Hospital Heping Fuyou Branch, Taipei 100, Taiwan.
5. Department of Applied Chemistry, National Pingtung University, Pingtung 900, Taiwan.
6. Nanofabrication Laboratory, Department of Electrical and Computer Engineering, University of Canterbury, New Zealand.
7. Center of Applied Nanomedicine, National Cheng Kung University, Tainan 70101, Taiwan.
8. Department of Urology, College of Medicine and Shu-Tien Urological Research Center, National Yang Ming Chiao Tung University, Taipei 112, Taiwan.
9. Department of Exercise and Health Sciences, University of Taipei, Taipei 100, Taiwan.
10. Core Facility Center, National Cheng Kung University, Tainan, 70101, Taiwan.

Prof. Fei-Ting Hsu

E-mail: sakiro920@mail.cmu.edu.tw

Prof. Yi-Chun Chiu

E-mail: DAM15@tpech.gov.tw

Prof. Chih-Chia Huang

E-mail: c2huang@email.ncku.edu.tw

Prof. Mei-Yi Liao

E-mail: myliao@mail.nptu.edu.tw

**Fig. S1.** EDS measurement of the elemental composition of the Fe_3_O_4_@Chl/Cu CNPs.

**Fig. S2.** Raman spectra of the Fe_3_O_4_@Chl/Fe CNPs synthesized with different Chl/Fe concentrations, capping-free Fe_3_O_4_ nanocrystals, and Chl/Fe molecules.

**Fig. S3.** (a) DLS, (b) zeta potential, and (c-f) the size distribution of cluster particles of Fe_3_O_4_@Chl/Fe CNPs synthesized with different Chl/Fe concentrations.

**Fig. S4.** SQUID measurement of Fe_3_O_4_@Chl/Fe_p[0-200 mg]_ CNPs at 300 K.

**Fig. S5.** (a) Photographs and (b-e) TEM images of Fe_3_O_4_ nanocrystal solutions with different citric acid concentrations. TEM images: (b) 2.1 mg (c) 10.6 mg (d) 21.2 mg (e) 42.4 mg.

**Fig. S6.** X-ray diffraction pattern of Fe_3_O_4_ nanocrystals synthesized by using different citrate acid concentrations.

**Fig. S7.** RNO/imidazole assay for the production of singlet oxygen species at 25 ppm_[Fe]_ from the Fe_3_O_4_@Chl/Fe CNPs, Fe_3_O_4_, Chl/Fe, and physically mixed Fe_3_O_4_+Chl/Fe molecules upon 660 nm light irradiation for 10 min.

**Fig. S8.** Thermal cycle curves of the Fe_3_O_4_@Chl/Fe_p[200 mg]_ CNPs ([Fe] = 100 ppm) under (a) 650 nm and (b) 808 nm laser irradiation. (c) Thermal cycle curves of Chl/Fe ([Chl/Fe] = 0.2 mM) under 650 nm and 808 nm laser irradiation.

**Fig. S9.** In vitro characterizations of the chromogenic performance of a variety of Fe_3_O_4_@Chl/Fe_[0-200 mg]_ CNPs ([Fe] = 0.5 ppm from AAS and [Chl/Cu] = 0.001 mM from UV–visible measurements). Fe_3_O_4_-N_2_H_4_ is the iron oxide from reference 45. (b) TMB assay for the reaction of iron-based materials at 0.5 ppm_[Fe]_ with 1 mM H_2_O_2_. (c) UV–vis spectra of Fe_3_O_4_@Chl/Fe_[200 mg]_ CNPs at 0.5 ppm_[Fe]_.

**Fig. S10.** TMB assay for evaluating the reaction of H_2_O_2_ with (a) 0.5 ppm_[Fe]_ Fe_3_O_4_@Chl/Fe_[200 mg]_ CNPs and 0.5 ppm_[Fe]_ ppm_[Fe]_ Fe_3_O_4_@Chl/Fe_[200 mg]_ CNPs plus 1 mM GSH and (b) 0.5 ppm_[Fe]_ Chl/Fe molecule and Chl/Fe molecule plus 1 mM GSH.

**Fig. S11.** GSH depletion assay under (a) 20 ppm_[Fe]_ Fe-based treatments and (b) 20 ppm_[Fe]_ Fe-based treatments combined with a 100 μM H_2_O_2_ solution.

**Fig. S12.** GSH depletion assay with 20 ppm Fe_3_O_4_@Chl/Fe_[200 mg]_ CNPs and Fe_3_O_4_@Chl/Fe_[200 mg]_-CPBA CNPs combined with irradiation by a 75 mW/cm^2^ 660 nm laser.

**Fig. S13.** AAS measurements for the uptake analysis of (a) T24 cancer cells, (b) MB49 cancer cells, (c) SV-HUC1 normal cells, and (d) Vero normal cells after treatment with Fe_3_O_4­_@Chl/Fe_[200 mg[_ and Fe_3_O_4­_@Chl/Fe_[200 mg[_-CPBA CNPs for 1, 16 and 24 h.

**Fig. S14.** Confocal images of T24 cells treated with Fe_3_O_4­_@Chl/Fe_[200 mg[_-CPBA CNPs. (a) Fluorescence and confocal images from bottom to top (b-d). (scale bar: 20 μm)

**Fig. S15.** Cell viability after treated with different concentrations of modified Fe_3_O_4­_@Chl/Fe_[200 mg[_ CNPs with targeting molecules such as CPBA, FA, RGD, and transferrin (a) without and (b) with a 660 nm LED light source are displayed.

**Fig. S16.** The cell viability of HeLa, NIH 3T3 and VERO cells treated with Fe_3_O_4­_@Chl/Fe_[200 mg]_ CNPs group and CPBA-, RGD-, and transferrin-conjugated Fe_3_O_4­_@Chl/Fe_[200 mg]_ CNPs without (a, c, e) and with (b, d, f) a 660 nm LED light source at 75 mW/cm^2^.

**Fig. S17.** The cell viability of MB49 treated with Fe_3_O_4_@Chl/Fe CNPs and Fe_3_O_4_@Chl/Fe-CPBA CNPs (a) without and (b) with irradiation by a 660 nm LED light source at 75 mW/cm^2^.

**Fig. S18.** The viability of T24 cells treated with Fe_3_O_4_@Chl/Fe_[5 mg and 200 mg]_ CNPs synthesized with 5 and 200 mg of Chl/Fe for 24 h. (a) Without light exposure (b) exposed to 660 nm LED light (75 mW/cm^2^) ([Fe] = 0.2, 1, 5, 10, 50, 100 ppm).

**Fig. S19.** Confocal images of MB49 cells treated with Fe_3_O_4_@Chl/Fe_[200 mg]_-CPBA CNPs after 24 h: (a) Fluorescence and confocal images from bottom to top (b-d). Scale bar: 20 μm.
